# Induction of labour at 39 weeks and adverse outcomes in low-risk pregnancies according to ethnicity, socioeconomic deprivation, and parity: A national cohort study in England

**DOI:** 10.1371/journal.pmed.1004259

**Published:** 2023-07-20

**Authors:** Patrick Muller, Amar M. Karia, Kirstin Webster, Fran Carroll, George Dunn, Alissa Frémeaux, Tina Harris, Hannah Knight, Sam Oddie, Asma Khalil, Jan Van Der Meulen, Ipek Gurol-Urganci

**Affiliations:** 1 Department of Health Services Research and Policy, London School of Hygiene & Tropical Medicine, London, United Kingdom; 2 Clinical Quality, Royal College of Obstetricians and Gynaecologists, London, United Kingdom; 3 Centre for Reproduction Research, Faculty of Health and Life Sciences, De Montfort University, Leicester, United Kingdom; 4 The Health Foundation, London, United Kingdom; 5 Bradford Neonatology, Bradford Teaching Hospitals NHS Foundation Trust, Bradford, United Kingdom; 6 Fetal Medicine Unit, Department of Obstetrics and Gynaecology, St George’s University Hospitals NHS Foundation Trust, London, United Kingdom; 7 Vascular Biology Research Centre, Molecular and Clinical Sciences Research Institute, St George’s University of London, London, United Kingdom; 8 Fetal Medicine Unit, Liverpool Women’s NHS Foundation Trust, Liverpool, United Kingdom

## Abstract

**Background:**

Ethnic and socioeconomic inequalities in obstetric outcomes are well established. However, the role of induction of labour (IOL) to reduce these inequalities is controversial, in part due to insufficient evidence. This national cohort study aimed to identify adverse perinatal outcomes associated with IOL with birth at 39 weeks of gestation (“IOL group”) compared to expectant management (“expectant management group”) according to maternal characteristics in women with low-risk pregnancies.

**Methods and findings:**

All English National Health Service (NHS) hospital births between January 2018 and March 2021 were examined. Using the Hospital Episode Statistics (HES) dataset, maternal and neonatal data (demographic, diagnoses, procedures, labour, and birth details) were linked, with neonatal mortality data from the Office for National Statistics (ONS). Women with a low-risk pregnancy were identified by excluding pregnancies with preexisting comorbidities, previous cesarean section, breech presentation, placenta previa, gestational diabetes, or a baby with congenital abnormalities. Women with premature rupture of membranes, placental abruption, hypertensive disorders of pregnancy, amniotic fluid abnormalities, or antepartum stillbirth were excluded only from the IOL group. Adverse perinatal outcome was defined as stillbirth, neonatal death, or neonatal morbidity, the latter identified using the English composite neonatal outcome indicator (E-NAOI). Binomial regression models estimated risk differences (with 95% confidence intervals (CIs)) between the IOL group and the expectant management group, adjusting for ethnicity, socioeconomic background, maternal age, parity, year of birth, and birthweight centile. Interaction tests examined risk differences according to ethnicity, socioeconomic background, and parity. Of the 1 567 004 women with singleton pregnancies, 501 072 women with low-risk pregnancies and with sufficient data quality were included in the analysis. Approximately 3.3% of births in the IOL group (1 555/47 352) and 3.6% in the expectant management group (16 525/453 720) had an adverse perinatal outcome. After adjustment, a lower risk of adverse perinatal outcomes was found in the IOL group (risk difference −0.28%; 95% CI −0.43%, −0.12%; *p* = 0.001). This risk difference varied according to socioeconomic background from 0.38% (−0.08%, 0.83%) in the least deprived to −0.48% (−0.76%, −0.20%) in the most deprived national quintile (*p*-value for interaction = 0.01) and by parity with risk difference of −0.54% (−0.80%, −0.27%) in nulliparous women and −0.15% (−0.35%, 0.04%) in multiparous women (*p*-value for interaction = 0.02). There was no statistically significant evidence that risk differences varied according to ethnicity (*p* = 0.19). Key limitations included absence of additional confounding factors such as smoking, BMI, and the indication for induction in the HES datasets, which may mean some higher risk pregnancies were included.

**Conclusions:**

IOL with birth at 39 weeks was associated with a small reduction in the risk of adverse perinatal outcomes, with 360 inductions in low-risk pregnancies needed to avoid 1 adverse outcome. The risk reduction was mainly present in women from more socioeconomically deprived areas and in nulliparous women. There was no significant risk difference found by ethnicity. Increased uptake of IOL at 39 weeks, especially in women from more socioeconomically deprived areas, may help reduce inequalities in adverse perinatal outcomes.

## Author summary

**Why was this study done?:**

- Adverse perinatal outcomes are more common among women from deprived areas and ethnic minorities in England.
- There is debate about whether induction of labour (IOL) with birth at 39 weeks should be offered based on ethnicity or deprivation in low-risk pregnancies, as a means of reducing inequalities.

**What did the researchers do and find?:**

- We analysed a database of all maternity admissions in the English National Health Service (NHS) and measured the association between IOL at 39 weeks and adverse perinatal outcomes in low-risk pregnancies.
- We found evidence of a small benefit from induction overall in low-risk pregnancies, with 360 inductions associated with the avoidance of 1 adverse perinatal outcome.
- The benefits of induction were observed mainly in women from more socioeconomically deprived areas and in nulliparous women.

**What do these findings mean?:**

- An increased uptake of IOL with birth at 39 weeks, especially in women from more socioeconomically deprived areas and in nulliparous women, may help reduce inequalities in perinatal outcomes.
- The greater benefit from IOL with birth at 39 weeks in women from more socioeconomically deprived areas may be explained by an increased prevalence of maternal risk factors.
- Improved collection of routine data on the indication for induction and the presence of risk factors is required to corroborate the role that IOL at 39 weeks in women with a low-risk pregnancy can play in reducing inequalities in risk of adverse perinatal outcomes.

## Introduction

In many high-income countries, the risk of adverse perinatal outcomes is markedly increased for women living in socioeconomically deprived areas and in women from minority ethnic groups [1–4]. In May 2021, the National Institute for Health and Care Excellence (NICE), a body publishing clinical practice guidelines for the English and Welsh National Health Service (NHS), circulated for consultation a draft of a national guideline on the use of induction of labour (IOL). It recommended the offer of IOL at 39 weeks of gestation for uncomplicated pregnancies if there were risk factors associated with maternal or neonatal complications. These included assisted conception, age 35 years or above, BMI of 30 kg/m^2^ or above, or being from a minority ethnic group [5].

This draft guideline was hotly debated, with many interest groups arguing that the recommendation to offer IOL at 39 weeks of gestation to women from a minority ethnic group was discriminatory, not evidence-based, and not addressing the root causes of differences in outcome by ethnicity [6]. Following the consultation, this recommendation was removed from the final guideline that was published in November 2021 [7].

A recent systematic review of randomised clinical trials of IOL at or beyond 37 weeks of gestation concluded that the optimal timing of IOL in women with low-risk pregnancies needs further research, especially for women with specific risk profiles [8]. A systematic review of observational studies found a significant reduction in the risk of neonatal intensive care admission and perinatal death with elective induction at 39 weeks compared to expectant management but did not explore outcomes in different populations [9].

In this study, we estimated the difference in the risk of adverse perinatal outcomes in low-risk women who had IOL with birth at 39 weeks of gestation or expectant management, using national population-based data of all births in the English NHS between 1 January 2018 and 31 March 2021.

A key feature of our study is that we used a set of exclusion criteria for the 2 comparison groups that aims to create a cohort of women who were all at low risk at 39 weeks. To do this, we excluded all women who had risk factors for adverse perinatal outcomes that are most likely to be present and known before 39 weeks (e.g., preexisting diabetes and hypertension, gestational diabetes, previous cesarean section, breech presentation).

In addition, we excluded women only from the group who had IOL and gave birth at 39 weeks if they had risk factors that can develop after 39 weeks (e.g., premature rupture of membranes, placental abruption, pregnancy-induced hypertension, pre-eclampsia, eclampsia, or amniotic fluid abnormalities), because if these risk factors are present at 39 weeks they are not compatible with expectant management. This design creates as closely as possible the appropriate comparison to evaluate differences in outcomes of women with a low-risk pregnancy who did and did not have IOL with birth at 39 weeks.

In response to the debate about the role of IOL in women from minority ethnic groups in the United Kingdom, we also tested if the differences in risk varied according to ethnicity and socioeconomic deprivation, because ethnicity and socioeconomic deprivation are tightly linked risk factors [10] as well as according to parity, because parity is a key determinant of adverse perinatal outcomes overall [10,11].

## Methods

### Ethics statement

This study used routinely collected administrative hospital data that were accessed to evaluate service provision and performance and was therefore exempt from ethical review by the NHS Health Research Authority. The use of personal data without individual consent was approved by the NHS Health Research Authority (16/CAG/0058).

### Data sources

Records of maternity episodes for women who had singleton births and the records of neonatal admissions in England between 1 January 2018 and 31 March 2021 were extracted from Hospital Episode Statistics (HES), an administrative database of all care episodes in English NHS hospitals. HES records include information on patient demographics, dates of admission and discharge, and on diagnoses, coded according to the ICD-10 system [12], and procedures, coded according to the OPCS-4 system [13]. Maternity records also capture labour and birth information in a series of data fields referred to as the “HES maternity tail” [14].

The women’s maternity records were linked to admission records of their babies using a mother–baby link file created by the Personal Demographics Service (PDS). Date of death was supplied by the Office for National Statistics (ONS) and linked to the neonatal admission records. In cases of conflicting information on gestational age at birth, the highest recorded in either the mother or baby record was used.

Exclusions were applied according to data completeness and quality criteria, following an approach developed for the National Maternity and Perinatal Audit (NMPA) and informed by earlier studies [15–17]. Women were excluded if their maternity records could not be linked to a neonatal record or lacked information on gestational age, birth status, or labour onset. Women were only included if we found that their NHS hospital had a rate of IOL between 10% and 50%, a stillbirth rate between 0.1% and 1.0%, and agreement of 70% or above between labour onset recorded as cesarean section and recorded delivery mode of cesarean section.

### Comparison groups

Gestational age at birth was only available in weeks. Two groups were defined: women with low-risk pregnancies with IOL and birth between 39 weeks and 0 days, and 39 weeks and 6 days (“IOL group”) and a group of women with low-risk pregnancies with a birth at 40 weeks or later (i.e., after 40 weeks and 0 days) with any mode of labour onset (“expectant management group”). This definition of the expectant management group follows definitions used in earlier studies [18,19]. It was chosen to ensure that we included only women in the expectant management group with a birth at a later gestation than in the IOL group.

We excluded all women who had pregnancies with conditions coded in the maternity records that are strongly associated with the probability of having IOL or adverse perinatal outcomes that are most likely present and known before 39 weeks: preexisting comorbidities, including diabetes, hypertension, cardiac or pulmonary disease, previous cesarean section, gestational diabetes, breech presentation, placenta previa, or congenital abnormalities (full list of ICD-10 codes in S1 Table).

In addition, we excluded women only from the group who had IOL and birth at 39 weeks if they had premature rupture of membranes, placental abruption, pregnancy-induced hypertension or pre-eclampsia, eclampsia, or amniotic fluid abnormalities. As explained in the Introduction, this creates a cohort of women with a low-risk pregnancy for whom the decision whether or not to recommend IOL at 39 weeks is relevant. Women who had IOL to deliver a baby who had died in utero (i.e., antepartum stillbirth) were also excluded from the group who had IOL at 39 weeks.

### Outcome definition

An adverse perinatal outcome was defined as a composite outcome including stillbirth, neonatal death within 28 days of birth, or presence of the English composite neonatal adverse outcome indicator (the E-NAOI) for liveborn babies [20]. The E-NAOI is a validated outcome based on the presence of any of 15 neonatal diagnoses or 7 procedures recorded in a baby’s admission record in HES, and it is highly associated with risk of further hospital admissions and death in the year following birth.

Stillbirth timing, either ante- or intrapartum, was recorded as “unknown” for 24% of stillbirths in the HES dataset. Certain diagnoses in the maternity record may indicate the timing of stillbirth in cases where it is recorded as “unknown,” so self-evident corrections were made to assign a timing in these cases (recoding rules in S2 Table). For example, if acidosis was recorded, the stillbirth timing was assumed to be intrapartum. In the primary analysis, stillbirths in the IOL group with unknown timing even after applying self-evident corrections were treated as antepartum (before the onset of labour) and therefore excluded.

### Statistical methods

**Fig 1:**
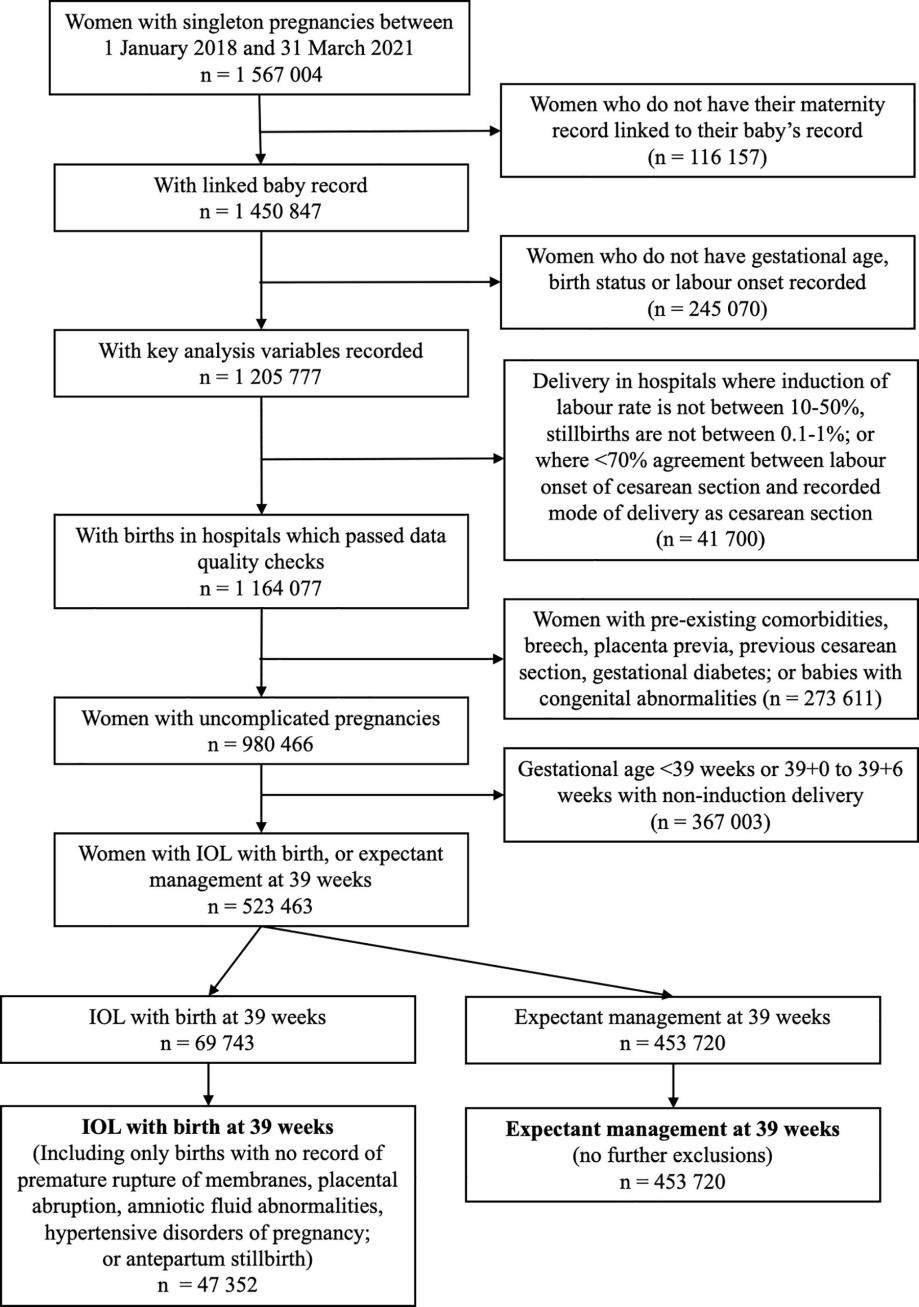
Flowchart showing impact of inclusion and exclusion criteria on number of women included at analysis.

A flow chart (Fig 1) was produced to show the impact of the different inclusion and exclusion criteria on the number of pregnancies eligible for analysis. Characteristics of the women and their pregnancies were summarised, and the frequency of adverse perinatal outcomes and frequency of each type were calculated.

Binomial regression models with an identity link function were fitted to estimate differences in risk of any adverse perinatal outcome associated with maternal characteristics and delivery [21]. Risk differences between the IOL and expectant management groups were calculated from models that adjusted for calendar year, maternal age, ethnicity (white, South Asian, black, mixed, or other ethnic group), parity (nulliparous or multiparous), socioeconomic deprivation according to national quintiles of the index of multiple deprivation (IMD) in lower layer super output areas (32 844 areas with a typical population between 1 000 and 3 000 people and between 400 and 1 200 households) [22], and birthweight centile (<10th, 10th to 89th, 90th to 100th). For all regression analyses, missing values for maternal age (<0.01%), ethnicity (12.4%), socioeconomic deprivation (0.7%), and birthweight centile (0.1%) were imputed using chained equations to generate 10 datasets. Estimates from these imputed datasets were pooled using Rubin’s rules.

The improvement to model fit, from the addition of interaction terms between IOL on the one hand and ethnicity, socioeconomic deprivation (national IMD quintile), or parity on the other hand was tested in separate models with likelihood ratio tests.

The number of IOLs needed to avoid 1 adverse perinatal outcome was also estimated using the adjusted risk differences and their 95% confidence intervals (95% CI). A negative number should be interpreted as the number of IOLs that is associated with 1 additional adverse perinatal outcome [23].

A *p*-value <0.05 was considered to indicate a statistically significant result. All analyses were done with Stata version 17, StataCorp, College Station, Texas, United States of America [21].

### Sensitivity analyses

Two sensitivity analyses were conducted. In the first sensitivity analysis, we tested the robustness of the results to only having information about the gestational age at birth in weeks. To do this, we expanded the definition of expectant management group and also included non-IOL births with a gestational age of 39 weeks (i.e., birth between 39 weeks and 0 days, and 39 weeks and 6 days), in addition to all births with a gestational age of 40 weeks or later (i.e., after 40 weeks and 0 days) by any mode of onset that were included in the primary analysis. In the second sensitivity analysis, we treated all stillbirths in the IOL group with unknown timing as intrapartum, whereas in the primary analysis, these were treated as antepartum and therefore removed from the analysis.

This study is reported as per the Strengthening the Reporting of Observational Studies in Epidemiology (STROBE) guideline (S1 STROBE Checklist). All analyses were conducted as originally planned, and the methods did not change in light of the results.

## Results

### Descriptive results

A total of 1 567 004 women with singleton pregnancies were recorded in the HES database between 1 January 2018 and 31 March 2021 (Fig 1). Of these, 402 927 women were excluded because their maternity admissions were not linked to neonatal admission records, key variables were not recorded, or they were admitted to hospital with low data quality. Of the 1 164 077 remaining women, 273 611 women were excluded because they did not have a low-risk pregnancy (see Methods), next 367 003 women were excluded because they had IOL and gave birth before 39 weeks or because they did not have IOL and gave birth before 40 weeks (see Methods). After applying the exclusion criteria only relevant for women who had IOL with birth at 39 weeks, a further 22 391 women were excluded. As a result, 501 072 women were included in the analysis (47 352 women who had IOL and gave birth at 39 weeks and 453 720 women in the expectant management group). The characteristics of those included and excluded due to data quality selection criteria were similar (S3 Table).

The 2 groups (IOL with birth at 39 weeks or expectant management) were similar with respect to ethnicity, national IMD quintile, and year of birth (Table 1). Women in the IOL group were more likely to be aged under 25 or over 40 years, to have a baby with a birthweight on the 90th centile or over than those in the expectant management group.

**Table 1:** IOL and expectant management at 39 weeks groups, according to maternal characteristics.

	IOL with birth at 39 weeks (*n* = 47 352)	Expectant management (*n* = 453 720)
**Age group**		
12–19 years	2 317 (4.89%)	14 774 (3.26%)
20–24	8 893 (18.78%)	67 865 (14.96%)
25–29	14 164 (29.91%)	134 853 (29.72%)
30–34	12 685 (26.79%)	152 655 (33.65%)
35–39	6 056 (12.79%)	73 080 (16.11%)
40+	3 237 (6.84%)	10 491 (2.31%)
Missing (% of total)	0 (0.00%)	2 (0.00%)
**Ethnicity**		
White	35 712 (83.30%)	319 174 (80.55%)
South Asian	3 590 (8.37%)	36 421 (9.19%)
Black	1 253 (2.92%)	15 089 (3.81%)
Mixed	788 (1.84%)	7 139 (1.80%)
Any other	1 526 (3.56%)	18 398 (4.64%)
Missing (% of total)	4 483 (9.47%)	57 499 (12.67%)
**Socioeconomic deprivation** **(national IMD quintile)**		
IMD Q1 = Least deprived	6 616 (14.05%)	74 570 (16.56%)
2	7 793 (16.55%)	81 195 (18.03%)
3	8 863 (18.82%)	89 212 (19.81%)
4	10 479 (22.26%)	99 802 (22.16%)
5 = Most deprived	13 332 (28.32%)	105 646 (23.45%)
Missing (% of total)	269 (0.57%)	3 295 (0.73%)
**Year of birth**		
2018	14 132 (29.84%)	162 845 (35.89%)
2019	15 261 (32.23%)	146 438 (32.27%)
2020	14 724 (31.09%)	119 681 (26.38%)
2021	3 235 (6.83%)	24 756 (5.46%)
**Birthweight centile**		
<10th	2 902 (6.13%)	29 656 (6.54%)
10th–89th	37 737 (79.73%)	386 352 (85.20%)
90th–100th	6 693 (14.14%)	37 433 (8.26%)
Missing (% of total)	20 (0.04%)	279 (0.06%)
**Parity**		
Nulliparous	21 750 (45.93%)	232 586 (51.26%)
Multiparous no previous cesarean birth	25 602 (54.07%)	221 134 (48.74%)

IMD, index of multiple deprivation; IOL, induction of labour.

The risk of any adverse perinatal outcome was 3.28% for the IOL group and 3.64% for the expectant management group (*p* < 0.001, Table 2). The types of adverse perinatal outcomes were also different. Pregnancies in the IOL group had a lower risk of stillbirth (0.01% versus 0.07%, *p* < 0.001) and other adverse perinatal outcome (3.18% versus 3.53%, *p* < 0.001) but a higher risk of neonatal death within 28 days (0.10% versus 0.04%, *p* < 0.001). The most common other adverse perinatal outcomes were ventilatory support (1.91% versus 2.33%) and respiratory distress syndrome (0.53% versus 0.77%, *p* < 0.001).

**Table 2:** Distribution of adverse perinatal outcomes for births with induction at 39 weeks or expectant management.

	IOL with birth at 39 weeks (*n* = 47 352)	Expectant management (*n* = 453 720)
Incidence of adverse perinatal outcomes* *n* (%)		
Adverse outcomes	1 555 (3.28%)	16 525 (3.64%)
Composition of adverse perinatal outcomes* *n* (%)		
Stillbirth	3 (0.01%)	337 (0.07%)
Neonatal death	46 (0.10%)	184 (0.04%)
Other adverse perinatal outcome	1 506 (3.18%)	16 004 (3.53%)
Frequency of different adverse perinatal outcomes** *n* (%)		
Birthweight < 1 500 g	9 (0.02%)	127 (0.03%)
Gestational age < 32 weeks	0 (0.00%)	0 (0.00%)
Respiratory distress syndrome	250 (0.53%)	3 507 (0.77%)
Seizure	36 (0.08%)	601 (0.13%)
Intraventricular haemorrhage (grades 3 or 4)	0 (0.00%)	16 (0.00%)
Cerebral infarction	2 (0.00%)	54 (0.01%)
Periventricular leukomalacia	0 (0.00%)	3 (0.00%)
Birth trauma	50 (0.11%)	501 (0.11%)
Hypoxic ischaemic encephalopathy	58 (0.12%)	880 (0.19%)
Necrotising enterocolitis	9 (0.02%)	30 (0.01%)
Sepsis/septicaemia	126 (0.27%)	947 (0.21%)
Pneumonia	76 (0.16%)	1 116 (0.25%)
Other respiratory outcomes	73 (0.15%)	775 (0.17%)
Chronic respiratory disease originating in perinatal period	1 (0.00%)	38 (0.01%)
Bacterial meningitis	19 (0.04%)	245 (0.05%)
Resuscitation	72 (0.15%)	942 (0.21%)
Ventilatory support (mechanical or CPAP)	905 (1.91%)	10 560 (2.33%)
Central venous or arterial catheter	210 (0.44%)	1 370 (0.30%)
Pneumothorax requiring intercostal catheter	76 (0.16%)	1 029 (0.23%)
Any IV fluids	62 (0.13%)	616 (0.14%)
Transfusion of blood or blood products	10 (0.02%)	18 (0.00%)
Any body cavity surgical procedure	213 (0.45%)	770 (0.17%)
Therapeutic hypothermia	52 (0.11%)	689 (0.15%)

* Adverse perinatal outcome: stillbirth, neonatal death and neonatal morbidity.

** A baby may have more than one of these conditions recorded so percentages add to more than 100%.

CPAP, continuous positive airway pressure; IOL, induction of labour.

### Modelling results

**Fig 2:**
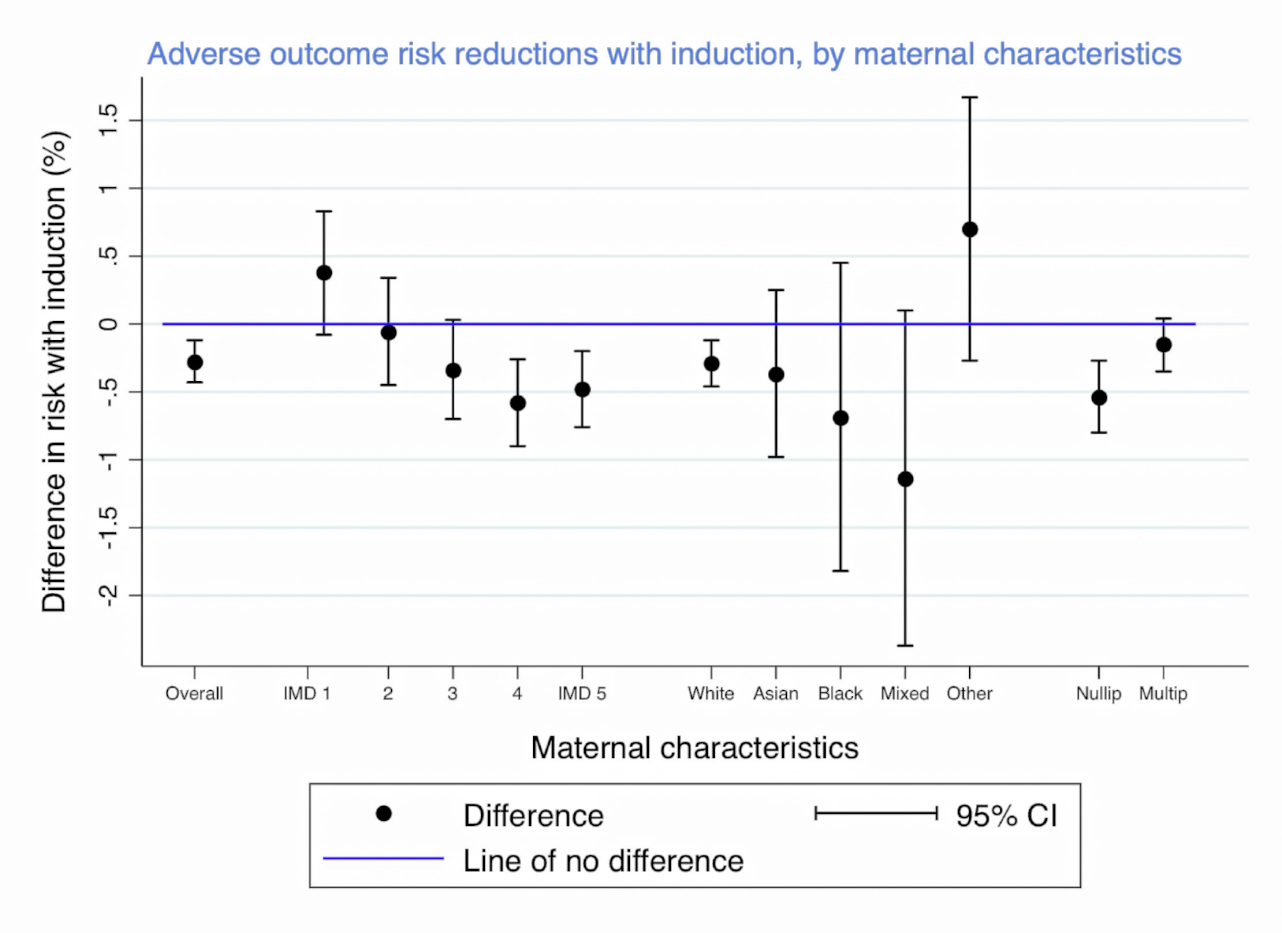
Difference in risk of adverse perinatal outcome with IOL at 39 weeks gestation compared to expectant management, by maternal characteristics. Results below 0% indicate lower risk with IOL, according to socioeconomic deprivation (in national IMD quintiles with 1 representing the least and 5 representing the most deprived quintile; see Methods) or ethnic group, and parity. Risk differences are shown with their 95% CI. CI, confidence interval; IMD, index of multiple deprivation; IOL, induction of labour.

In the fully adjusted binomial regression model, the IOL group had a lower risk of adverse perinatal outcome with a risk difference of −0.28% (95% CI: −0.43%, −0.12%), compared to the expectant management group (Table 3). This difference varied according to the women’s socioeconomic background (*p*-value for interaction = 0.01; full results in S4 Table). For women in the most deprived quintiles (IMD 4 and 5), there was a significant reduction in the risk of adverse perinatal outcome (risk difference −0.58% (−0.90%, −0.26%) and −0.48% (−0.76%, −0.20%), respectively). For women in the least deprived quintile (IMD 1), there was no statistically significant difference between the IOL and the expectant management group (0.38% (−0.08%, 0.83%)) (Fig 2). There was no statistically significant evidence that the differences in risk of adverse perinatal outcome between the IOL and the expectant management group varied according to ethnicity (*p*-value for interaction = 0.19). The risk reduction with IOL and birth at 39 weeks was greater in nulliparous women −0.54% (−0.80%, −0.27%) than in multiparous women −0.15% (−0.35%, 0.04%) (*p*-value for interaction = 0.02; full results in S4 Table) (Fig 2).

**Table 3:** Differences in risk of adverse perinatal outcome with induction at 39 weeks (IOL) compared to expectant management, where negative risk difference indicates lower risk with IOL. Risk differences are presented from unadjusted and adjusted models, and also from models with interaction terms allowing the risk differences to be different between women with different characteristics (deprivation, ethnicity, or parity).

	Risk difference between IOL with birth at 39 weeks and expectant management (95% CI)	*P*-value for risk difference	Number of IOLs to avoid 1 adverse perinatal outcome (95% CI) **
**Unadjusted model**	−0.36% (−0.53%, −0.19%)	<0.001	279 (189, 530)
Fully adjusted model* without any interactions	−0.28% (−0.43%, −0.12%)	0.001	360 (230, 832)
Fully adjusted model* with additional interaction term between socioeconomic deprivation and IOL; separate risk differences are presented for each deprivation group (*p*-value for interaction = 0.01)			
IMD Q1 = Least deprived	0.38% (−0.08%, 0.83%)	0.11	-
2	−0.06% (−0.45%, 0.34%)	0.78	-
3	−0.34% (−0.70%, 0.03%)	0.07	-
4	−0.58% (−0.90%, −0.26%)	<0.001	173 (111, 389)
5 = Most deprived	−0.48% (−0.76%, −0.20%)	0.001	210 (132, 505)
Fully adjusted model* with additional interaction term between ethnicity and IOL; separate risk differences are presented for each ethnic group (*p*-value for interaction = 0.19)			
White	−0.29% (−0.46%, −0.12%)	0.001	349 (219, 863)
South Asian	−0.37% (−0.98%, 0.25%)	0.24	-
Black	−0.69% (−1.82%, 0.45%)	0.24	-
Mixed	−1.14% (−2.37%, 0.10%)	0.07	-
Any other	0.70% (−0.27%, 1.67%)	0.16	-
Fully adjusted model* with additional interaction term between parity and IOL; separate risk differences are presented for each parity group (*p*-value for interaction = 0.02)			
Nulliparous	−0.54% (−0.80%, −0.27%)	<0.001	186 (125, 365)
Multiparous	−0.15% (−0.35%, 0.04%)	0.13	-

* The adjusted model includes maternal age, ethnicity, socioeconomic deprivation, year of delivery, birthweight centile, and parity.

** Only reported if the CI for risk difference does not cross 0. A negative number corresponds to a negative risk difference, i.e., more adverse perinatal outcomes with IOL.

CI, confidence interval; IOL, induction of labour.

According to the results from the fully adjusted analysis, the number of inductions at 39 weeks associated with avoiding 1 adverse perinatal outcome was estimated at 360 (= 1/−0.277%; 95% CI: 230, 832). In the model with a deprivation interaction, this number for women in the most deprived quintile was estimated to be lower at 210 (= 1/−0.477%; 95% CI: 132, 505) and with a parity interaction, the number for nulliparous women was 186 (= 1/−0.537%; 95% CI: 125, 365).

### Sensitivity analyses

When the expectant management group was expanded to also include women with non-IOL births at 39 weeks, there was no longer evidence for an overall difference between the groups in the fully adjusted model (S5 Table), with a risk difference of −0.08% (95% CI: −0.23%, 0.08%; *p* = 0.34). However, the risk differences persisted according to socioeconomic background (*p*-value for interaction = 0.004), with a higher risk of an adverse perinatal outcome with IOL at 39 weeks in the least deprived quintile (IMD 1, risk difference: 0.60%, 95% CI: 0.15%, 1.05%) and a lower risk with IOL at 39 weeks in the more deprived quintiles (risk differences: −0.33%, 95% CI: −0.65%, −0.01%, and −0.33%, 95% CI: −0.61%, −0.06%, IMD 4 and 5, respectively). The results from second sensitivity analysis, where stillbirths with unknown timing were considered intrapartum instead of antepartum, was similar to the primary analysis following adjustment (risk difference: −0.26% (−0.42%, −0.10%), *p* = 0.001).

## Discussion

To our knowledge, our results provide the largest comparison of IOL with birth at 39 weeks with expectant management in women with low-risk singleton pregnancies to date. We found that women who were induced and gave birth at 39 weeks had lower risk of adverse perinatal outcomes compared to women who had expectant management and gave birth after 39 weeks. However, the difference was small and we estimate that overall 360 inductions are needed to avoid 1 adverse perinatal outcome.

The evidence of a difference in risk was observed especially in women from more socioeconomically deprived areas and in nulliparous women. There was no statistically significant evidence that the reduction in risk associated with IOL at 39 weeks varied according to ethnic group.

Our results add to previous analyses of the effect of IOL with birth at 39 weeks on perinatal outcomes in women with low-risk term pregnancies in Scotland and England that used routinely collected data sources with maternity records linked to baby records [18–20]. The results of both the Scottish study, including women of all ages, and the English study, including women aged 35 years or over, suggested that IOL at 40 weeks may reduce overall rates of perinatal death.

The systematic review of randomised control trials of routine IOL found that only 8 of the 34 trials included had examined IOL before 40 weeks [8]. The largest and most recent of these trials randomised 6 096 low-risk nulliparous women to either IOL at 39 weeks or expectant management and reported a reduction in a composite outcome, including perinatal death and severe neonatal complications, from 5.4% to 4.3% [24]. The systematic review of observational studies found results that were broadly consistent with these trial findings, for example, reporting a 20% reduction in the risk of neonatal intensive care unit admissions, pooling 3 studies that together compared 23 647 women who had elective IOL at 39 weeks of gestation with 164 771 women who had expectant management [9].

Our study found a much smaller reduction in the risk of adverse perinatal outcomes with IOL at 39 weeks of gestation. One explanation for this smaller reduction is that we used a different composite adverse outcome measure. Another explanation is that we studied a population of women at lower risk, given that we included both nulliparous and multiparous women. The latter explanation is corroborated by the observation that the risk of perinatal mortality is 0.11% (521 deaths in 453 720 births) in the expectant management group in our study, whereas the corresponding risk is 0.20% (284 deaths in 140 184 births) in the systematic review of observational studies [9]. However, further explanations, including residual confounding because of incomplete risk adjustment as well as incomplete identification of IOL and adverse outcomes, cannot be excluded.

An important feature of our study is that we could include more than 500 000 women with a low-risk pregnancy that allowed the use of statistical interaction tests to assess if the differences in risk between the IOL and expectant management groups at 39 weeks varied according to ethnicity, socioeconomic deprivation, and parity. It is important to note that even in our study, including low-risk pregnancies over a 3-year period, the statistical power to detect variation in the differences in risk associated with IOL at 39 weeks is small. For example, only 5% of pregnant women in England are from black and 12% from South Asian ethnic backgrounds [25].

It is likely that the adjustment of the difference in risk between women who had IOL at 39 weeks and those who had expectant management will have been incomplete. Firstly, the routinely collected data that we analysed did not include the indication for induction of labour. Secondly, some risk factors were imperfectly or not adjusted for in our analysis. For example, data on maternal risk factors such as BMI and smoking and fetal factors such as congenital anomalies are not collected in HES. Also, preexisting maternal comorbidities, including hypertension and diabetes, are likely to be under-ascertained that implies that some women were erroneously included in our cohort, which may have led to an overestimation of the risk of adverse pregnancy outcomes in low-risk pregnancies [26].

Our data period includes births affected by the Coronavirus Disease 2019 (COVID-19) pandemic. We previously demonstrated a 1% increase in induction of labour rates with no increase in stillbirth rates compared to pre-pandemic periods [27]. It is therefore unlikely that the COVID-19 pandemic has had a substantial impact on the results observed. Our analysis excluded records missing data on key variables and hospitals with data of poor quality. This may have introduced bias, but it is unlikely to have affected our results in a major way given that the characteristics were similar between the initial and final cohorts.

A final limitation is that the date of IOL is not available in the routinely collected data that we used. Therefore, gestational age at birth had to be used as a proxy measure for the timing of IOL, thereby introducing a small degree of crossover between the comparison groups, because a number of women who gave birth at 40 weeks will have been induced at 39 weeks, which will have led to a slight underestimation of the actual risk difference between IOL and expectant management.

The first sensitivity analysis in which we expanded the expectant management group by including women with non-IOL births at 39 weeks, found that the overall difference in risk between the IOL at 39 weeks and the expectant management group disappeared. However, women from the most socioeconomically deprived areas still seemed to have a lower risk of adverse perinatal outcome with IOL at 39 weeks. There is a suggestion of increased risk difference following IOL at 39 weeks for women in the least deprived quintile. However, differences in the indications for IOL and uncoded risk factors may underlie the observed differences according to socioeconomic deprivation. The other sensitivity analysis demonstrated that our results were robust to uncertainties about the timing of stillbirth (i.e., antepartum or intrapartum).

What does our study mean for recommendations on IOL at 39 weeks of gestation for women with an uncomplicated singleton pregnancy? It shows that IOL with birth at 39 weeks is associated with a small reduction in the risk of adverse perinatal outcomes overall which is in line with previous randomised and observational studies. However, the question to what extent IOL at 39 weeks of gestation should be recommended especially for a number of specific groups as originally proposed in the draft guideline proposed by NICE for the English and Welsh NHS needs careful consideration. We found evidence that the reduction in risk associated with IOL at 39 weeks is mainly present in women from more socioeconomically deprived areas and in nulliparous women. The reduction in risk associated with IOL at 39 weeks was also greater in women from a minority ethnic group, but the corresponding statistical test of interaction did not produce a statistically significant result, however—as explained earlier—these should be interpreted with caution because of low statistical power.

In summary, more comprehensive data are required to further explore the pathways associated with ethnicity, socioeconomic deprivation, parity, and risk of adverse perinatal outcomes. Better coding of risk factors, indications for, and decision-making around the offer of IOL should further inform our understanding of obstetric risk patterns. Based on existing evidence and the results of our study, we conclude that increased uptake of IOL at 39 weeks may help reduce socioeconomic inequalities in perinatal outcomes.

## Supporting information

S1 STROBE Checklist STROBE checklist. (DOC)

S1 Table Definition of inclusion criteria and case-mix conditions using Hospital Episode Statistics (HES) database. (DOCX)

S2 Table Recoding rules for assigning a stillbirth timing where it is not recorded. (DOCX)

S3 Table Impact of data quality related selection criteria on distribution of demographic characteristics of women included. (DOCX)

S4 Table Binomial regression model with identity link function for risk of adverse perinatal outcome. (DOCX)

S5 Table Sensitivity analyses results. (DOCX)

## Abbreviations

CI: confidence interval
COVID-19: Coronavirus Disease 2019
HES: Hospital Episode Statistics
IMD: index of multiple deprivation
IOL: induction of labour
NHS: National Health Service
NICE: National Institute for Health and Care Excellence
NMPA: National Maternity and Perinatal Audit
ONS: Office for National Statistics
PDS: Personal Demographics Service

## References

[1] Manktelow BN, Smith LK, Seaton SE, Hyman-Taylor P, Kurinczuk JJ, Field DJ, et al. MBRRACE-UK Perinatal Mortality Surveillance Report, UK Perinatal Deaths for Births from January to. Leicester: The Infant Mortality and Morbidity Studies, Department of Health Sciences, University of Leicester. December 2014:2016.

[2] Draper ES, Gallimore ID, Smith LK, Fenton AC, Kurinczuk JJ, Smith PW, et al. MBRRACE-UK Perinatal Mortality Surveillance Report, UK Perinatal Deaths for Births from January to. Leicester: The Infant Mortality and Morbidity Studies, Department of Health Sciences, University of Leicester. December 2018:2020.

[3] Bryant AS, Worjoloh A, Caughey AB, Washington E. Racial/ethnic disparities in obstetric outcomes and care: prevalence and determinants. Am J Obstet Gynecol. 2010;202(4):335–343. doi: 10.1016/j.ajog.2009.10.864 20060513 PMC2847630

[4] Flenady V, Wojcieszek AM, Middleton P, Elwood D, Erwich JJ, Coory M, et al. Stillbirths: recall to action in high-income countries. Lancet. 2016;387(10019):691–702. doi: 10.1016/S0140-6736(15)01020-X 26794070

[5] National Institute for Health and Care Excellence. Guideline: Inducing Labour, draft for consultation, May 2021. London, UK; 2021. Available from: https://www.nice.org.uk/guidance/ng207/documents/draft-guideline-2, accessed 2023 May 26.

[6] Mahase E. Doctors question NICE recommendation to induce labour at 39 weeks in ethnic minority women. BMJ. 2021;374:n1711. doi: 10.1136/bmj.n1711 34230033

[7] National Institute for Health and Care Excellence. Inducing labour: NICE guideline NG207. 2021. Available from: https://www.nice.org.uk/guidance/ng207, accessed 2023 May 26.

[8] Middleton P, Shepherd E, Morris J, Crowther CA, Gomersall JC. Induction of labour at or beyond 37 weeks’ gestation. Cochrane Database Syst Rev. 2020;7(7):Cd004945. doi: 10.1002/14651858.CD004945.pub5 32666584 PMC7389871

[9] Grobman WA, Caughey AB. Elective induction of labor at 39 weeks compared with expectant management: a meta-analysis of cohort studies. Am J Obstet Gynecol. 2019;221(4):304–310. doi: 10.1016/j.ajog.2019.02.046 30817905

[10] Jardine J, Walker K, Gurol-Urganci I, Webster K, Muller P, Hawdon J, et al. Adverse pregnancy outcomes attributable to socioeconomic and ethnic inequalities in England: a national cohort study. Lancet. 2021;398(10314):1905–1912. doi: 10.1016/S0140-6736(21)01595-6 34735797

[11] Jardine J, Blotkamp A, Gurol-Urganci I, Knight H, Harris T, Hawdon J, et al. Risk of complicated birth at term in nulliparous and multiparous women using routinely collected maternity data in England: cohort study. BMJ. 2020;371:m3377. doi: 10.1136/bmj.m3377 33004347 PMC7527835

[12] World Health Organisation. ICD-10 Version: 2010. 2010. Available from: https://icd.who.int/browse10/2010/en, accessed 2023 May 26.

[13] NHS Digital. OPCS Classification of Interventions and Procedures. 2021. Available from: https://datadictionary.nhs.uk/supporting_information/opcs_classification_of_interventions_and_procedures.html, accessed 2023 May 26.

[14] Herbert A, Wijlaars L, Zylbersztejn A, Cromwell D, Hardelid P. Data Resource Profile: Hospital Episode Statistics Admitted Patient Care (HES APC). Int J Epidemiol. 2017;46(4):1093–93i. doi: 10.1093/ije/dyx015 28338941 PMC5837677

[15] NMPA Project Team. National Maternity and Perinatal Audit: Clinical Report 2019. Based on births in NHS maternity services between 1 April 2016 and 31 March 2017. London: RCOG; 2019.

[16] Ghosh RE, Ashworth DC, Hansell AL, Garwood K, Elliot P, Toledano MB. Routinely collected English birth data sets: comparisons and recommendations for reproductive epidemiology. Arch Dis Child Fetal Neonatal Ed. 2016;101(5):F451–F457. doi: 10.1136/archdischild-2015-309540 26837309

[17] Murray J, Saxena S, Modi N, Majeed A, Aylin P, Bottle A, et al. Quality of routine hospital birth records and the feasibility of their use for creating birth cohorts. J Public Health (Oxf). 2013;35(2):298–307. doi: 10.1093/pubmed/fds077 22967908

[18] Knight HE, Cromwell DA, Gurol-Urganci I, Harron K, van der Meulen JH, Smith GCS. Perinatal mortality associated with induction of labour versus expectant management in nulliparous women aged 35 years or over: An English national cohort study. PLoS Med. 2017;14(11):e1002425. doi: 10.1371/journal.pmed.1002425 29136007 PMC5685438

[19] Stock SJ, Ferguson E, Duffy A, Ford I, Chalmers J, Norman JE. Outcomes of elective induction of labour compared with expectant management: population based study. BMJ. 2012;344:e2838. doi: 10.1136/bmj.e2838 22577197 PMC3349781

[20] Knight HE, Oddie SJ, Aughey HKL, van der Meulen JH, Gurol-Urganci I, Cromwell DA. Establishing a composite neonatal adverse outcome indicator using English hospital administrative data. Arch Dis Child Fetal Neonatal Ed. 2019;104(5):F502–F509. doi: 10.1136/archdischild-2018-315147 30487299 PMC6703994

[21] StataCorp. 2021. Stata: Release 17. Statistical Software. College Station, TX: StataCorp LLC.

[22] English indices of deprivation 2015. Ministry of Housing, Communities & Local Government. 2015. Available from: https://www.gov.uk/government/statistics/english-indices-of-deprivation-2015, accessed 2023 May 26.

[23] Cook RJ, Sackett DL. The number needed to treat: a clinically useful measure of treatment effect. BMJ. 1995;310(6977):452–454. doi: 10.1136/bmj.310.6977.452 7873954 PMC2548824

[24] Grobman WA, Rice MM, Reddy UM, Tita ATN, Silver RM, Mallett G, et al. Labor Induction versus Expectant Management in Low-Risk Nulliparous Women. NEJM. 2018;379(6):513–523. doi: 10.1056/NEJMoa1800566 30089070 PMC6186292

[25] Webster K, NMPA Project Team. Ethnic and Socio-economic Inequalities in NHS Maternity and Perinatal Care for Women and their Babies: Assessing care using data from births between 1 April 2015 and 31 March 2018 across England, Scotland and Wales. London: RCOG; 2021.

[26] NMPA Project Team. National Maternity and Perinatal Audit: Clinical Report 2022. Based on births in NHS maternity services in England and Wales between 1 April 2018 and 31 March 2019. London: RCOG; 2022.

[27] Gurol-Urganci I, Waite L, Webster K, Jardine J, Carroll F, Dunn G, et al. Obstetric interventions and pregnancy outcomes during the COVID-19 pandemic in England: A nationwide cohort study. PLoS Med. 2022;19(1):e1003884. doi: 10.1371/journal.pmed.1003884 35007282 PMC8803187

