# Supplementary material for: Induction of labour at 39 weeks and adverse outcomes in low-risk pregnancies according to ethnicity, socioeconomic deprivation, and parity: A national cohort study in England
Source: PLoS Med. 2023 Jul 20;20(7):e1004259. doi: 10.1371/journal.pmed.1004259 (PMC10358943; doi:10.1371/journal.pmed.1004259)
Supplement: S1 Table — (DOCX) [file pmed.1004259.s002.docx]

**S1 Table: Definition of inclusion criteria using Hospital Episodes Statistics (HES) database**

| **Condition** | **Use in analysis** | **ICD10/OPCS4 codes, or variables used to identify condition in HES admission records** |
| --- | --- | --- |
| Pre-existing diabetes mellitus | Exclusion criteria | ICD10: E10-E11, E13-E14; O24.0-O24.3 |
| Pre-existing hypertension | Exclusion criteria | ICD10: I10-I15, O10, O11 |
| Pulmonary disease | Exclusion criteria | ICD10: J (excluding J45: asthma), I26, I27, I28 |
| Cardiac disease | Exclusion criteria | ICD10: I00-I02, I05-I09, I2, I31, I4, I51-I52, I6, I7 |
| Previous caesarean section | Exclusion criteria | Identified using mode of birth OPCS4 codes in historical maternity records |
| Breech presentation | Exclusion criteria | ICD10: O32.1, O64.1, O32.0, O32.2, O80.1, O83.0, O83.1  OPCS4: R19, R20 |
| Gestational diabetes | Exclusion criteria | ICD10: O24.4 |
| Placenta previa | Exclusion criteria | ICD10: O44 |
| Congenital abnormalities | Exclusion criteria | ICD10: Q00-Q99 |
|  |  |  |
| Premature rupture of membranes | Excluded from IOL group only | ICD10: O42 |
| Placental abruption | Excluded from IOL group only | ICD10: O45 |
| Amniotic fluid abnormalities | Excluded from IOL group only | ICD10: O41.1, O40 |
| Hypertensive disorders of pregnancy | Excluded from IOL group only | ICD10: O13, O14, O15, O16 |
| Antepartum stillbirth | Excluded from IOL group only | Stillbirth identified using birth status variable and ICD10 code Z37.1    Timing of stillbirth identified using birth status variable and if unknown or missing, with further recoding to antepartum/intrapartum using ICD10 codes (Supplementary Table S2) |
