# Supplementary material for: Induction of labour at 39 weeks and adverse outcomes in low-risk pregnancies according to ethnicity, socioeconomic deprivation, and parity: A national cohort study in England
Source: PLoS Med. 2023 Jul 20;20(7):e1004259. doi: 10.1371/journal.pmed.1004259 (PMC10358943; doi:10.1371/journal.pmed.1004259)
Supplement: S2 Table — (DOCX) [file pmed.1004259.s003.docx]

**S2 Table: Recoding rules for assigning a stillbirth timing where it is not recorded.**

| **ICD-10 code** | **Description** | **Recoding assigned** |
| --- | --- | --- |
| O60.3 | Preterm delivery without spontaneous labour | Stillbirth (unknown timing) recoded to stillbirth (antepartum) |
| Z35.1 | Supervision of pregnancy with history of abortive outcome | Stillbirth (unknown timing) recoded to stillbirth (antepartum) |
| O35.1 | Maternal care for (suspected) chromosomal abnormality in fetus, not applicable or unspecified | Stillbirth (unknown timing) recoded to stillbirth (antepartum) |
| Z35.4 | Supervision of pregnancy with grand multiparity | Stillbirth (unknown timing) recoded to stillbirth (antepartum) |
| O41.8 | Other specified disorders of amniotic fluid and membranes, | Stillbirth (unknown timing) recoded to stillbirth (antepartum) |
| O32.2 | Maternal care for transverse and oblique lie, not applicable or unspecified | Stillbirth (unknown timing) recoded to stillbirth (antepartum) |
| O14.2 | HELLP syndrome (HELLP), unspecified trimester | Stillbirth (unknown timing) recoded to stillbirth (antepartum) |
| O44.1 | Complete placenta previa with haemorrhage, unspecified trimester | Stillbirth (unknown timing) recoded to stillbirth (antepartum) |
| O69.4 | Labour and delivery complicated by vasa previa, not applicable or unspecified | Stillbirth (unknown timing) recoded to stillbirth (antepartum) |
| O82.0 | Delivery by elective caesarean section | Stillbirth (unknown timing) recoded to stillbirth (antepartum) |
| O02.1 | Missed abortion | Stillbirth (unknown timing) recoded to stillbirth (antepartum) |
| O04.6 | Delayed or excessive haemorrhage following (induced) termination of pregnancy | Stillbirth (unknown timing) recoded to stillbirth (antepartum) |
| O68.0 | Labour and delivery complicated by fetal heart rate anomaly | Stillbirth (unknown timing) recoded to stillbirth (intrapartum) |
| O45.9 | Premature separation of placenta, unspecified, unspecified trimester | Stillbirth (unknown timing) recoded to stillbirth (intrapartum) |
| O35.8 | Maternal care for other (suspected) fetal abnormality and damage, not applicable or unspecified | Stillbirth (unknown timing) recoded to stillbirth (intrapartum) |
| O68.2 | Labour and delivery complicated by fetal heart rate anomaly with meconium in amniotic fluid | Stillbirth (unknown timing) recoded to stillbirth (intrapartum) |
| O75.8 | Maternal exhaustion complicating labour and delivery | Stillbirth (unknown timing) recoded to stillbirth (intrapartum) |
| O42.1 | Premature rupture of membranes, onset of labour more than 24 hours following rupture | Stillbirth (unknown timing) recoded to stillbirth (intrapartum) |
| O42.0 | Premature rupture of membranes, onset of labour within 24 hours of rupture | Stillbirth (unknown timing) recoded to stillbirth (intrapartum) |
| O68.8 | Labour and delivery complicated by other evidence of fetal stress | Stillbirth (unknown timing) recoded to stillbirth (intrapartum) |
| O69.0 | Labour and delivery complicated by prolapse of cord, not applicable or unspecified | Stillbirth (unknown timing) recoded to stillbirth (intrapartum) |
| O60.1 | Preterm labour with preterm delivery, unspecified trimester, not applicable or unspecified | Stillbirth (unknown timing) recoded to stillbirth (intrapartum) |
| O32.6 | Maternal care for compound presentation, not applicable or unspecified | Stillbirth (unknown timing) recoded to stillbirth (intrapartum) |
| O32.3 | Maternal care for face, brow and chin presentation, not applicable or unspecified | Stillbirth (unknown timing) recoded to stillbirth (intrapartum) |
| O62.4 | Hypertonic, incoordinate, and prolonged uterine contractions | Stillbirth (unknown timing) recoded to stillbirth (intrapartum) |
| E87.2 | Acidosis | Stillbirth (unknown timing) recoded to stillbirth (intrapartum) |
| O62.8 | Other abnormalities of forces of labour | Stillbirth (unknown timing) recoded to stillbirth (intrapartum) |
| O68.3 | Labour and delivery complicated by biochemical evidence of fetal stress | Stillbirth (unknown timing) recoded to stillbirth (intrapartum) |
| O64.1 | Obstructed labour due to breech presentation, not applicable or unspecified | Stillbirth (unknown timing) recoded to stillbirth (intrapartum) |
