# Supplementary material for: Induction of labour at 39 weeks and adverse outcomes in low-risk pregnancies according to ethnicity, socioeconomic deprivation, and parity: A national cohort study in England
Source: PLoS Med. 2023 Jul 20;20(7):e1004259. doi: 10.1371/journal.pmed.1004259 (PMC10358943; doi:10.1371/journal.pmed.1004259)
Supplement: S3 Table — (DOCX) [file pmed.1004259.s004.docx]

**S3 Table: Impact of data quality related selection criteria on distribution of demographic characteristics of women included.**

|  | **Before any exclusions**  **(n = 1 567 004)** | **After excluding women who did not have link to baby's record**  **(n = 1 450 847)** | **After excluding women who did not have gestational age, birth status or labour onset all recorded**  **(n = 1 205 777)** | **Including only births in hospitals which passed data quality checks ***  **(n = 1 164 077)** |
| --- | --- | --- | --- | --- |
| **Age group** | | | | |
| 12-19 years | 46 235 (3.0%) | 43 583 (3.0%) | 36 558 (3.0%) | 35 512 (3.1%) |
| 20-24 | 213 528 (13.6%) | 199 008 (13.7%) | 166 753 (13.8%) | 161 582 (13.9%) |
| 25-29 | 432 795 (27.6%) | 402 071 (27.7%) | 336 457 (27.9%) | 325 469 (28.0%) |
| 30-34 | 516 640 (33.0%) | 477 845 (32.9%) | 396 810 (32.9%) | 382 773 (32.9%) |
| 35-39 | 291 515 (18.6%) | 267 462 (18.4%) | 219 878 (18.2%) | 211 434 (18.2%) |
| 40+ | 66 281 (4.2%) | 60 872 (4.2%) | 49 315 (4.1%) | 47 301 (4.1%) |
| *Missing (% of total)* | *10 (0.0%)* | *6 (0.0%)* | *6 (0.0%)* | *6 (0.0%)* |
| **Ethnicity** | | | | |
| White | 1 068 815 (76.9%) | 995 768 (77.4%) | 832 468 (77.8%) | 802 554 (77.7%) |
| South Asian | 163 724 (11.8%) | 149 541 (11.6%) | 124 097 (11.6%) | 120 322 (11.7%) |
| Black | 63 424 (4.6%) | 55 289 (4.3%) | 44 784 (4.2%) | 42 784 (4.1%) |
| Mixed | 28 032 (2.0%) | 24 807 (1.9%) | 20 270 (1.9%) | 19 349 (1.9%) |
| Any other | 65 256 (4.7%) | 60 662 (4.7%) | 49 008 (4.6%) | 47 654 (4.6%) |
| *Missing (% of total)* | *177 753 (11.3%)* | *164 780 (11.4%)* | *135 150 (11.2%)* | *131 414 (11.3%)* |
| **Socioeconomic deprivation (National IMD quintile)** | | | | |
| IMD Q1 = Least deprived | 234 533 (15.1%) | 218 314 (15.1%) | 183 474 (15.3%) | 176 739 (15.3%) |
| 2 | 266 820 (17.1%) | 247 366 (17.2%) | 203 965 (17.0%) | 196 837 (17.0%) |
| 3 | 301 724 (19.4%) | 279 070 (19.4%) | 230 326 (19.2%) | 221 245 (19.1%) |
| 4 | 348 101 (22.4%) | 320 732 (22.3%) | 268 602 (22.4%) | 260 051 (22.5%) |
| 5 = Most deprived | 405 184 (26.0%) | 375 578 (26.1%) | 311 116 (26.0%) | 300 976 (26.0%) |
| *Missing (% of total)* | *10 642 (0.7%)* | *9 787 (0.7%)* | *8 294 (0.7%)* | *8 229 (0.7%)* |
| **Parity** | | | | |
| Nulliparous | 693 418 (49.6%) | 642 422 (49.6%) | 527 850 (48.8%) | 510 399 (48.8%) |
| Multiparous, no previous caesarean birth | 705 922 (50.4%) | 652 953 (50.4%) | 554 443 (51.2%) | 534 664 (51.2%) |
| **Birthweight centile** | | | | |
| <10^th^ | 93 411 (6.1%) | 87 738 (6.1%) | 72 774 (6.0%) | 69 689 (6.0%) |
| 10^th^ – 89^th^ | 1 251 583 (81.3%) | 1 177 053 (81.2%) | 979 571 (81.3%) | 945 269 (81.3%) |
| 90^th^ – 100^th^ | 194 378 (12.6%) | 184 627 (12.7%) | 152 217 (12.6%) | 147 923 (12.7%) |
| *Missing (% of total)* | *27 632 (1.8%)* | *1 429 (0.1%)* | *1 215 (0.1%)* | *1 196 (0.1%)* |
| **Year of birth** | | | | |
| 2018 | 550 360 (35.1%) | 503 967 (34.7%) | 427 587 (35.5%) | 413 181 (35.5%) |
| 2019 | 513 696 (32.8%) | 474 817 (32.7%) | 392 652 (32.6%) | 379 457 (32.6%) |
| 2020 | 418 134 (26.7%) | 390 354 (26.9%) | 320 139 (26.6%) | 308 587 (26.5%) |
| 2021 | 84 814 (5.4%) | 81 709 (5.6%) | 65 399 (5.4%) | 62 852 (5.4%) |
| * Rates of induction of labour between 10-50%; stillbirths between 0.1% and 1%; > 70% agreement between labour onset of caesarean section and recorded delivery mode of caesarean section. | | | | |
