# Supplementary material for: Induction of labour at 39 weeks and adverse outcomes in low-risk pregnancies according to ethnicity, socioeconomic deprivation, and parity: A national cohort study in England
Source: PLoS Med. 2023 Jul 20;20(7):e1004259. doi: 10.1371/journal.pmed.1004259 (PMC10358943; doi:10.1371/journal.pmed.1004259)
Supplement: S4 Table — (DOCX) [file pmed.1004259.s005.docx]

**S4 Table: Binomial regression model with identity link function for risk of adverse perinatal outcome.**

|  | **Full model with interaction term between IOL and IMD** | | **Full model with interaction term between IOL and Parity** | | |
| --- | --- | --- | --- | --- | --- |
| **Characteristic** | **Risk difference (95% confidence interval)** | **P-value** | **Risk difference (95% confidence interval)** | **P-value** |  |
| **Induction** | | | | | |
| Expectant management | Reference |  | Reference |  |  |
| Induction at 39 weeks | 0.38% (-0.08%, 0.83%) | 0.11 | -0.54% (-0.80%, -0.27%) | <0.001 |  |
| **Age group** | | | | | |
| 12-19 years | Reference |  | Reference |  |  |
| 20-24 | 0.31% (0.01%, 0.60%) | 0.04 | 0.32% (0.02%, 0.61%) | 0.04 |  |
| 25-29 | 0.19% (-0.09%, 0.47%) | 0.19 | 0.19% (-0.09%, 0.47%) | 0.18 |  |
| 30-34 | 0.21% (-0.08%, 0.49%) | 0.15 | 0.21% (-0.08%, 0.49%) | 0.15 |  |
| 35-39 | 0.46% (0.16%, 0.75%) | 0.003 | 0.46% (0.16%, 0.75%) | 0.003 |  |
| 40+ | 0.39% (0.00%, 0.78%) | 0.05 | 0.41% (0.01%, 0.80%) | 0.04 |  |
| **Ethnicity** | | | | | |
| White | Reference |  |  |  |  |
| South Asian | 0.66% (0.48%, 0.85%) | <0.001 | 0.67% (0.48%, 0.85%) | <0.001 |  |
| Black | 1.48% (1.16%, 1.81%) | <0.001 | 1.49% (1.17%, 1.81%) | <0.001 |  |
| Mixed | 0.67% (0.24%, 1.10%) | 0.002 | 0.68% (0.25%, 1.10%) | 0.002 |  |
| Any other | 0.11% (-0.14%, 0.37%) | 0.39 | 0.11% (-0.14%, 0.37%) | 0.39 |  |
| **Socioeconomic deprivation (National IMD quintile)** | | | | | |
| IMD Q1 = Least deprived | Reference |  |  |  |  |
| 2 | 0.005% (-0.16%, 0.17%) | 0.96 | -0.04% (-0.20%, 0.13%) | 0.67 |  |
| 3 | 0.19% (0.02%, 0.36%) | 0.03 | 0.12% (-0.04%, 0.28%) | 0.14 |  |
| 4 | 0.18% (0.01%, 0.34%) | 0.04 | 0.08% (-0.07%, 0.24%) | 0.30 |  |
| 5 = Most deprived | -0.03% (-0.19%, 0.14%) | 0.74 | -0.12% (-0.27%, 0.04%) | 0.14 |  |
| **Socioeconomic deprivation & induction interaction** | | | | | |
| induction # IMD Q1 | Reference |  | n/a | n/a |  |
| induction # 2 | -0.43% (-1.04%, 0.17%) | 0.16 | n/a | n/a |  |
| induction # 3 | -0.71% (-1.30%, -0.13%) | 0.02 | n/a | n/a |  |
| induction # 4 | -0.95% (-1.51%, -0.40%) | 0.001 | n/a | n/a |  |
| induction # 5 | -0.85% (-1.39%, -0.32%) | 0.002 | n/a | n/a |  |
| **Parity** | | | | | |
| Nulliparous | Reference |  | Reference |  |  |
| Multiparous | -1.67% (-1.77%, -1.57%) | <0.001 | -1.71% (-1.81%, -1.60%) | <0.001 |  |
| **Parity & induction interaction** | | | | | |
| induction # nulliparous | n/a | n/a | Reference |  |  |
| induction # multiparous | n/a | n/a | 0.38% (0.06%, 0.71%) | 0.02 |  |
| **Birthweight centile** | | | | | |
| <10^th^ | Reference |  | Reference |  |  |
| 10^th^ – 89^th^ | -1.38% (-1.61%, -1.15%) | <0.001 | -1.37% (-1.61%, -1.14%) | <0.001 |  |
| 90^th^ – 100^th^ | 3.91% (3.57%, 4.25%) | <0.001 | 3.92% (3.58%, 4.26%) | <0.001 |  |
| **Year** | | | | | |
| 2018 | Reference |  | Reference |  |  |
| 2019 | -0.03% (-0.15%, 0.08%) | 0.59 | -0.03% (-0.15%, 0.08%) | 0.57 |  |
| 2020 | -0.16% (-0.28%, -0.04%) | 0.01 | -0.16% (-0.28%, -0.04%) | 0.01 |  |
| 2021 | -0.37% (-0.59%, -0.16%) | 0.001 | -0.37% (-0.58%, -0.16%) | 0.001 |  |
