# Supplementary material for: Induction of labour at 39 weeks and adverse outcomes in low-risk pregnancies according to ethnicity, socioeconomic deprivation, and parity: A national cohort study in England
Source: PLoS Med. 2023 Jul 20;20(7):e1004259. doi: 10.1371/journal.pmed.1004259 (PMC10358943; doi:10.1371/journal.pmed.1004259)
Supplement: S5 Table — (DOCX) [file pmed.1004259.s006.docx]

**S5 Table:** **Sensitivity analyses results**

Differences in risk of adverse perinatal outcome associated with IOL compared to expectant management.

|  | **Risk difference (95% CI)** | **P-value** | **Number of IOLs to avoid one adverse perinatal outcome**  **(95% CI) *** | **Interaction p-value** |
| --- | --- | --- | --- | --- |
| **Fully adjusted model without interaction** | |  |  |  |
| *Expectant management including other (non-IOL) births at 39 weeks* | -0.08% (-0.23%, 0.08%) | 0.34 | - | N/A |
| *Assume stillbirths with unknown timing were intrapartum instead* | -0.26% (-0.42%, -0.10%) | 0.001 | 383 (239, 968) | N/A |
| **Fully adjusted model and interaction with socioeconomic deprivation (national IMD quintile)** | | | | |
| *Expectant management including other (non-IOL) births at 39 weeks* | | | | 0.004 |
| IMD Q1 = Least deprived | 0.60% (0.15%, 1.05%) | 0.01 | -167 (-689, -95) |  |
| 2 | 0.14% (-0.25%, 0.54%) | 0.48 | - |  |
| 3 | -0.12% (-0.48%, 0.24%) | 0.52 | - |  |
| 4 | -0.33% (-0.65%, -0.01%) | 0.04 | 304 (154, 10718) |  |
| 5 = Most deprived | -0.33% (-0.61%, -0.06%) | 0.02 | 301 (165, 1706) |  |
| *Assume stillbirths with unknown timing were intrapartum instead* | | | | 0.01 |
| IMD Q1 = Least deprived | 0.41% (-0.05%, 0.87%) | 0.08 | - |  |
| 2 | -0.05% (-0.45%, 0.34%) | 0.79 | - |  |
| 3 | -0.33% (-0.70%, 0.03%) | 0.07 | - |  |
| 4 | -0.55% (-0.87%, -0.22%) | 0.001 | 183 (115, 450) |  |
| 5 = Most deprived | -0.46% (-0.74%, -0.18%) | 0.001 | 216 (135, 547) |  |
| **Fully adjusted model and interaction with parity (national IMD quintile)** | | | | |
| *Expectant management including other (non-IOL) births at 39 weeks* | | | | 0.13 |
| Nulliparous | -0.24% (-0.50%, 0.02%) | 0.07 | - |  |
| Multiparous | 0.01% (-0.19%, 0.20%) | 0.94 | - |  |
| *Assume stillbirths with unknown timing were intrapartum instead* | | | | 0.02 |
| Nulliparous | -0.52% (-0.78%, -0.25%) | <0.001 | - |  |
| Multiparous | -0.13% (-0.33%, -0.06%) | 0.18 | - |  |

* Only reported if the CI for risk difference does not cross 0. A negative number corresponds to a negative risk difference, i.e. more adverse perinatal outcomes with IOL.
